# Kinases and Cancer

**DOI:** 10.3390/cancers10030063

**Published:** 2018-03-01

**Authors:** Jonas Cicenas, Egle Zalyte, Amos Bairoch, Pascale Gaudet

**Affiliations:** 1 Department of Microbiology, Immunology and Genetics, Max F. Perutz Laboratories, University of Vienna, 1030 Vienna, Austria; 2 Proteomics Center, Institute of Biochemistry, Vilnius University Life Sciences Center, Sauletekio al. 7, LT-10257 Vilnius, Lithuania; egle.zalyte@gmail.com; 3 MAP Kinase Resource, Bioinformatics, Melchiorstrasse 9, 3027 Bern, Switzerland; 4 CALIPHO Group, SIB Swiss Institute of Bioinformatics, 1 rue Michel-Servet, CH-1211 Geneva 4, Switzerland; Amos.Bairoch@sib.swiss; 5 Faculty of Medicine; University of Geneva; 1 rue Michel-Servet, CH-1211 Geneva 4, Switzerland

Protein kinases are a large family of enzymes catalyzing protein phosphorylation. The human genome contains 518 protein kinase genes, 478 of which belong to the classical protein kinase family and 40 are atypical protein kinases. Phosphorylation is one of the critical mechanisms for regulating different cellular functions, such as proliferation, cell cycle, apoptosis, motility, growth, differentiation, among others. Deregulation of kinase activity can result in dramatic changes in these processes. Moreover, deregulated kinases are frequently found to be oncogenic and can be central for the survival and spread of cancer cells [1]. There are several ways for kinases to become involved in cancers: mis-regulated expression and/or amplification, aberrant phosphorylation, mutation, chromosomal translocation, and epigenetic regulation. 

The CALIPHO group of the SIB Swiss Institute of Bioinformatics develops neXtProt, a knowledge base focused on human proteins [2]. CALIPHO has fully annotated 300 of the best characterized protein kinases with respect to their normal function and their role in disease and pathogenesis. It has generated a corpus of around 30,000 statements about each of these proteins. This data is being progressively integrated into the neXtProt database. As of February 2018, neXtProt has integrated the GO biological processes annotations captured in the framework of this project, representing both the signaling pathways in which each kinase is implicated, as well as the role of those pathways in higher level processes, such as apoptosis, cellular proliferation, and development. These functions may give insights into the mechanisms of pathogenesis of each different kinase. This first set of annotation comprises approximately 5000 different statements, extracted from over 5000 publications. In the neXtProt web interface, this data can be identified by the ‘Source’ set as neXtProt in the right-hand side of the annotation table visible on the neXtProt page for each entry (https://www.nextprot.org/) (see Figure 1). 

Two datasets remain to be integrated: the first contains approximately 11,000 annotations describing the substrates and phosphorylation sites of these substrates, which can provide valuable insight to identify potential drug targets, or, importantly, predict undesirable side effects. 

The second dataset contains close to 6000 manually extracted annotations of great interest for cancer researchers: the potential use of each kinase as biomarkers (prognostic, diagnostic, or predictive); any reported misregulation of expression in disease, at the mRNA and/or protein, or resulting from aberrant epigenetic regulation; genetic variants associated with diseases, and finally, the use of the protein kinase as a disease model. 

Kinase amplifications could be used as diagnostic, prognostic, and predictive biomarkers in various cancer types. The best examples of kinase gene amplifications could be EGFR in non-small cell lung [3], colorectal [4], bladder [5] pancreatic [6], and breast [7] cancers; ERBB2 in breast [8], esophageal [9], gastric [10], and ovarian cancers [11]; MET in on-small cell lung [12], gastric [13], and colorectal cancers [14]; and AKT2 in pancreatic [15] and ovarian cancers [16]. Similarly, overexpression of mRNA or protein kinases are very well known in cancers and used as biomarker. Again, EGFR [17], ERBB2 [18], EPHA2 [19], and AKT2 [20] could be a good example. 

The phosphorylation of some kinases, such as EGFR [21,22], ERBB2 [21,23,24], ERK [25], AURKA [26], p38 [27], and AKT [28,29] is associated with prognosis in cancers and, in some cases, is a better marker than expression of the kinase. In addition, the substrates of kinases are known to be biomarkers in various cancers. For example SCH1 [30], p21Cip1 [31], p27Kip1 [32], androgen receptor [33], and retinoblastoma protein (RB) [34] have been shown to be prognostic biomarkers in breast and pancreatic cancer. 

One of the most extreme paths to the cancer development and progression is the mutations of the various genes, including kinases. The mutated kinases can become constitutively active and thus cause diverse cellular anomalies, leading to cancer initiation or growth. Probably the most well-known mutated kinase is BRAF, which is frequently mutated on Val-600 (p.V600E) [35,36] and is a driver mutation in several cancers, including colorectal cancer [37], melanoma [37], thyroid cancer [38] and non-small cell lung cancer [39]. Other frequent mutations occur in KIT [40], EGFR [41], and FTL3 [42]. 

Chromosomal translocations can also be cancer drivers. The most well-known translocation creates what is known as the Philadelphia chromosome, it is a translocation that creates a fusion of BCR with the ABL1 tyrosine kinase fusion with BCR and the subsequent constitutive activation of the kinase. Around 95% of patients with chronic myelogenous leukemia have this abnormality [43], as well as 25% with acute lymphoblastic leukemia [44]. Another famous translocation is EML4-ALK, first detected in lung adenocarcinomas and later found in different types of lung cancers [45]. FIP1L1-PDGFRA is another example of kinase translocation, resulting in eosinophilias and leukemias [46]. 

One of the fields for a better understanding of cancer biology is epigenetics, which includes modifications in chromatin structure through DNA chemical alteration, post-translational modifications of DNA bound proteins as well as gene expression regulation through non-coding RNAs, the processes all of which are involved in tumorigenesis and metastatic predisposition. Some kinases have been shown to be regulated by epigenetic mechanisms, such as RET [46,47], AATK [48], EPHA5 [49], CHK2 [50], and PKD1 [51]. 

Because of the pivotal function of kinases in cell biology and their role in numerous cancers, an intensive search for kinase inhibitors both for research purposes and for therapeutic usage has been ongoing for several decades. The first inhibitor, which provided the proof of principle that abnormal kinase inhibition can be used for cancer therapy, was imatinib (Gleevec), an inhibitor of ABL1 as well as the BCR-ABL1 fusion protein [52] (Figure 2). Several families of kinases, such as tyrosine kinases [53], cycle-dependent kinases [54,55,56], aurora kinases [57,58], mTOR [59], and mitogen-activated protein kinases [60] have already have FDA approved inhibitors and/or inhibitors, which are at different phases of clinical trials. Another approach to inhibit receptor tyrosine kinase signaling is the use of monoclonal antibodies. Trastuzumab (Herceptin), which targets ERBB2, was the first US Food and Drug Administration-approved anti-receptor tyrosine kinase monoclonal antibody [61]. There are also approved antibodies for EGFR [62], VEGFR2 [63], and PDGFR [64]. 

In summary, this special issue of Cancers is a collection of basic, translational, and clinical research articles as well as reviews, discussing the major impact of protein kinases, signaling pathways regulated by these enzymes and inhibitors of kinases on cancer biology and therapy. 

## Figures and Tables

**Figure 1:**
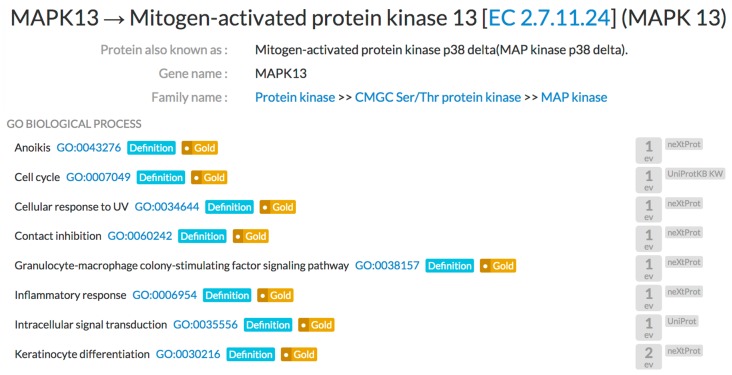
The neXtProt function page for MAPK13 (https://www.nextprot.org/entry/NX_O15264/).

**Figure 2:**
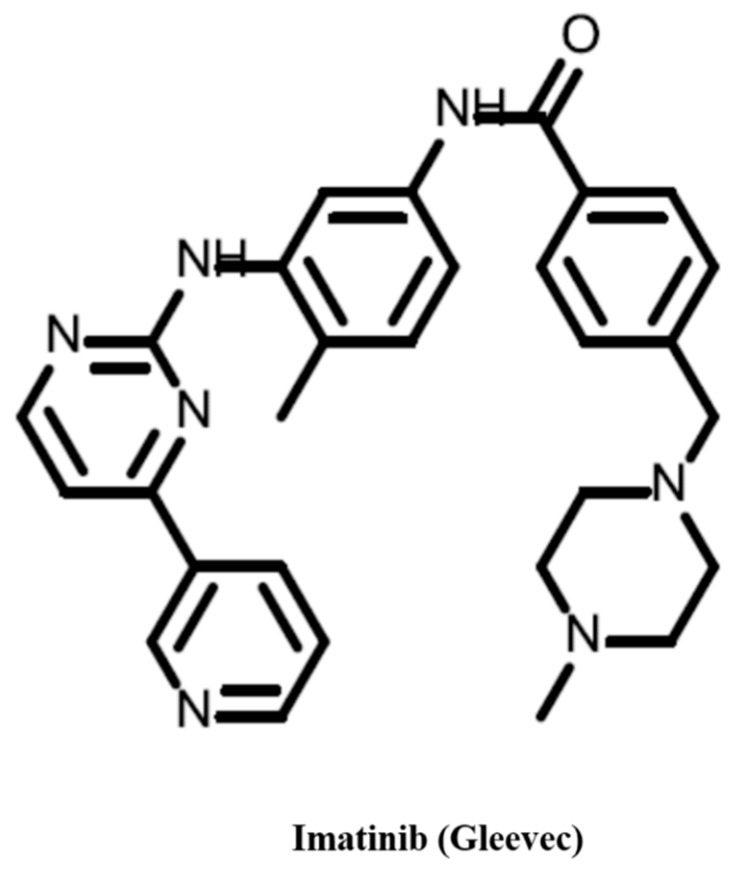
Imatinib (Gleevec) is the first FDA approved kinase inhibitor. Approved for the treatment of KIT+ GIST and Ph+ CML.

## Data Availability

All annotations are available at the neXtProt website (https://www.nextprot.org), as well as in XML from the ftp site (ftp://ftp.nextprot.org/pub/current_release/), via our API (https://api.nextprot.org/), and by query our SPARQL endpoint (https://sparql.nextprot.org/).
